# hsa_circ_0007376 Promotes Gastric Cancer Proliferation and Malignant Metastasis by Enhancing the Stability of IGF2BP3

**DOI:** 10.5152/tjg.2025.24491

**Published:** 2025-05-20

**Authors:** LinHu Liang, Ting Han, ZhengRong Zhang, ZhengWu Cheng, HaoRan Li

**Affiliations:** Department of Gastrointestinal Surgery, The First Affiliated Hospital of Wannan Medical College, Anhui, China

**Keywords:** Gastric cancer, hsa_circ_0007376, IGF2BP3, malignancy, proliferation

## Abstract

**Background/Aims:**

This study investigated the action of hsa_circ_0007376 in promoting the proliferation and metastasis of gastric cancer (GC).

**Materials and Methods:**

hsa_circ_0007376 was detected in GC tissues and cells by quantitative reverse transcription polymerase chain reaction. RNase R digestion, nucleoplasmic separation, and actinomycin D assays were conducted to detect the presence of hsa_circ_0007376 and its cyclic nature. The tumor-promoting effect of hsa_circ_0007376 in GC cells was verified by CCK-8, colony formation, wound healing, and Transwell assays. An interplay between hsa_circ_0007376 and insulin-like growth factor 2 mRNA binding protein 3 (IGF2BP3) was confirmed by FISH, RIP, and RNA pull-down experiments. The function of hsa_circ_0007376 on GC proliferation and metastasis was evaluated in vivo in a GC xenograft mouse model.

**Results:**

hsa_circ_0007376 was highly expressed in GC. hsa_circ_0007376 was associated with lymphatic metastasis, Tumor node metastasis (TNM) stage, and tumor size in GC. When hsa_circ_0007376 was knocked down, GC cells were prevented from proliferating, migrating, and invading, as well as being prevented from metastasizing. hsa_circ_0007376 was able to bind to IGF2BP3, thereby promoting GC.

**Conclusion:**

hsa_circ_0007376 may play a role in GC by interacting and enhancing the stability of the IGF2BP3 protein.

**Main Points**

- hsa_circ_0007376 knockdown represses proliferation and migration of gastric cancer cells.
- hsa_circ_0007376 binds to insulin-like growth factor 2 mRNA binding protein 3 in gastric cancer cells.
- Insulin-like growth factor 2 mRNA binding protein 3 overexpression reverses the inhibition of proliferation and metastasis of gastric cancer cells by downregulating hsa_circ_0007376.
- hsa_circ_0007376 promotes gastric cancer metastasisin vivo.

## Introduction

Gastric cancer (GC), a frequent malignant tumor of the gastrointestinal system, is the fourth most prevalent cause of cancer-related mortality worldwide.^1^^,^^2^ Even with advancements in clinical diagnosis and treatment approaches, individuals with GC face unfavorable prognoses and elevated death rates, attributed to frequent recurrences, distant metastases, and the absence of reliable early diagnostic markers.^3^ The only radical treatment for GC is surgery, and radiotherapy or other therapies are often used as adjuvants to surgery.^4^ The molecular mechanisms of GC development, therefore, need to be studied in more detail in order to identify new diagnostic markers and therapeutic targets.

CircRNAs are RNAs that have a closed-loop structure and are incapable of coding for proteins.^5^ The presence of circulatory RNAs has been linked to human diseases, including cancer, as well as potential molecular markers for diagnosing and treating these diseases.^6^ Different circRNAs are aberrantly expressed in digestive tract tumors, including GC, lung cancer, liver cancer, and colorectal cancer.^7^^,^^8^ Researchers have found that aberrant circulating RNA expression not only contributes to cancer cell proliferation but also to invasion, migration, and metastasis. For example, circRNA_100290 increases the proliferation and invasion of GC cells.^9^ Gastric cancer cells are prevented from proliferating, migrating, and invading when circNRIP1 is knocked down.^10^ hsa_circ_0007376 is a cyclic RNA originating from MAP2K2, and circRNA_102415, which is homologous to MAP2K2, has been shown to have upregulated expression levels in GC tissues.^11^ In GC, however, no research has explored hsa_circ_0007376’s mechanism of action.

Cytoplasmically localized circRNAs are much more stable than linear RNAs. circRNAs have a tertiary structure with greater protein adsorption capacity. CircRNA-interacting RBPs (RNA binding protein) are involved in target gene synthesis, translation, transcriptional regulation, and extracellular translocation.^12^ Aside from their role in RNA metabolism, RBPs are also involved in tumorigenesis and tumor development, making them potential targets for cancer treatment.^13^^,^^14^ Insulin-like growth factor 2 mRNA binding protein 3, also known as IGF2BP3, is a mammalian protein that binds IGF2 mRNA. The IGF2BP3 gene is ubiquitously expressed in eukaryotic tissues, and it is frequently upregulated in cancer cells in humans.^15^ The biological functions of IGF2BP3 in cancer are typically related to mRNA splicing, stability, and translational regulation.^16^ Research has shown that IGF2BP3 regulates cancer progression by interacting with noncoding RNAs.^17^^,^^18^ Insulin-like growth factor 2 mRNA binding protein 3 acts as a cancer-promoting agent in GC, enhancing the proliferation and migratory activities of GC cells.^19^ In GC cells, IGF2BP3 can bind to circFNDC3B to promote cellular migration and invasion.^20^ circARID1A binds to IGF2BP3 to promote cancer proliferation.^21^ Nevertheless, the mechanism of IGF2BP3 carcinogenesis in GC is not clearly understood.

This study identified hsa_circ_0007376 as a novel oncogene in GC, noting its upregulation in GC tissues and association with metastasis. Mechanistically, hsa_circ_0007376 directly interacts with IGF2BP3, which promotes GC cell proliferation and increases cell migration.

## Materials and Methods

### Clinical Sampling

GC tissues and adjacent tissues were collected from 50 patients undergoing surgical resection for GC at the hospital. Before surgery or biopsy, none of the GC patients received chemotherapy or radiotherapy. A rapid freezer was used to store the tissue samples after resection at −80°C. The study was approved by the Ethics Committee of The First Affiliated Hospital of Wannan Medical College Hospital (Approval no. 202201AH-05; approval date: January 5, 2022). All enrolled patients signed an informed consent form. Table 1 provides clinical information about the patients.

### Cell Culture

Normal gastric epithelial cell line (GES-1) and GC cell lines (MKN45, HGC-27, and MKN7) were sourced from ATCC (Manassas, USA). All cells were cultured in RPMI 1640 medium (GIBCO BRL, NY, USA) supplemented with 10% FBS (fetal bovine serum) (Invitrogen, USA) and 1% penicillin/streptomycin (Invitrogen) under 5% CO_2_ at 37°C.

### Cell Transfection

The small interfering RNA targeting hsa_circ_0007376 (si-hsa_circ_0007376), the negative control (si-NC), the IGF2BP3 overexpression plasmid pcDNA3.1-IGF2BP3, and the control plasmid (pcDNA-NC) were supplied by Ribobio (Guangzhou, China) and were correctly sequenced. The indicated nucleotides or plasmids were transfected into cells using Lipofectamine 3000 (Invitrogen, Carlsbad, CA). The multiplicity of infection was set at 100. Subsequently, stable transfected cell lines were screened using puromycin. Finally, quantitative reverse transcription polymerase chain reaction (RT-qPCR) was performed to measure transfection efficiency.

### Subcellular Localization of CircRNA

MKN45 cells, once immersed in a cytoplasmic lysis buffer, underwent centrifugation. After extracting the cytoplasmic supernatant, the surviving cells were incubated and centrifuged using lysis buffer. Subsequently, the TRIzol reagent (TaKaRa) facilitated the extraction of RNA from both the nucleus and cytoplasm. Quantitative reverse transcription polymerase chain reaction was conducted to identify hsa_circ_0007376, U6 (nucleus control), and glyceraldehyde 3-phosphate dehydrogenase (GAPDH) (cytoplasm control).

### RNase R Experiment

Total RNA in MKN45 was isolated using TRIzol reagent (Invitrogen). Incubation of total RNA (2 μg) at 37°C for 15 minutes with or without 3 U/mg RNase R (Epicentre Technologies, USA) was conducted. hsa_circ_0007376 and its corresponding linear gene MAP2K2 were detected by RT-qPCR.

### ActD Assay

MKN45 cells were exposed to 5 mg/mL ActD (Sigma-Aldrich) at 0, 4, 8, 12, and 24 hours. Cells were then harvested, and total RNA was extracted. hsa_circ_0007376 stability was analyzed using RT-qPCR.

### Cell Counting kit-8 (CCK-8)

MKN45 cells were harvested 48 hours after transfection, inoculated in 96-well plates (1 × 10^3^ cells/well), and routinely cultured. Each well was supplemented with 10 μL of CCK-8 solution (Dojindo, Kumamoto, Japan) and incubated for 2 hours at hours 0, 24, 48, and 72, respectively. At the end, the 96-well plate was placed in a microtiter reader (Bio-Tek, US) and the absorbance was measured at 450 nm.

### Colony Formation

Each 6.0-cm Petri dish was covered with 1 × 103 MKN-45 cells at 37°C with 5% CO_2_. Following a fortnight, the medium was removed, the colonies were meticulously cleaned twice with PBS, and stained for 20 minutes with crystal violet. Counting colonies was done with a microscope (Nikon, Tokyo, Japan).

### Wound Healing

MKN-45 cells were grown to 90% confluence and then linearly scraped on the cell monolayers. The scratched areas were imaged with an inverted microscope (Olympus) at 0 and 24 hours post-scratch. An analysis of the wound healing percentage was performed by measuring the distance of the scratch.

## Transwell

In the cell migration test, MKN-45 cells (1 × 10^5^ cells) were grown in a Transwell upper chamber (BD Biosciences, USA). During the cellular invasion examination, Matrigel (2 mg/mL, BD Biosciences) was utilized for pre-layering the inserts. Cells in the upper compartment were grown in a medium devoid of serum. A cell culture medium with 10% FBS was introduced into the lower chamber. After a 24-hour period, the cells, either invaded or migrated, were stabilized in methanol, dyed with 0.1% crystal violet (Solarbio), visualized, and tallied.

### RNA Pull Down

Biotin-labeled hsa_circ_0007376 and its antisense sequence were synthesized by GenePharma (Shanghai, China). The hsa_circ_0007376 probe, tagged with biotin, was lysed and incubated with MKN-45 cells. Western blotting was used to assess the cell lysates after they had been incubated at room temperature with streptavidin-conjugated agarose magnetic beads (Thermo Fisher Scientific).

### RNA immunoprecipitation (RIP)

RIP tests were performed with Millipore’s Magna RIP Kit. After lysing MKN-45 cells in RIP lysis solution for 30 minutes on ice, the cells were centrifuged and then co-incubated for 6 hours at 4°C with magnetic beads conjugated to either IgG (#02-6102, Invitrogen) or Ago2 (#ab32381, Abcam, UK). After eluting the immunoprecipitates from the bound beads using an elution buffer, RT-qPCR was conducted to quantify IGF2BP3 and hsa_circ_0007376.

### Fluorescence in situ Hybridization (FISH)

MKN-45 cells underwent fixation in 4% paraformaldehyde for a quarter-hour, followed by a PBS rinse and subsequent treatment with pepsin (1%, 10 mM HCl). The cells were hybridized with a Cy3-labeled hsa_circ_0007376 probe at 37°C overnight, dehydrated, and re-stained with 4'-6-diamidino-2-phenylindole (DAPI) before observations under a confocal laser microscope.

### Quantitative Reverse Transcription Polymerase Chain Reaction

Trizol (Invitrogen) was employed to isolate total RNA from GC cells or tumor tissues. Using the PrimeScript^TM^ RT kit (TaKaRa), 1 μg of total RNA was reverse transcribed to cDNA. TB Green^TM^ Premix Ex TaqTM II (TaKaRa) was employed in polymerase chain reaction (PCR), which was carried out using an ABI 7500 real-time fluorescence quantitative PCR machine (Applied Biosystems, USA). GAPDH served as the reference point. The target gene expression was computed using the 2^−ΔΔCT^ technique. Table 2 presents the primer sequences.

### Western Blot

Using radio immunoprecipitation assay (RIPA) buffer enriched with protease inhibitors (Beyotime, Shanghai, China), proteins were isolated from cells and tumors. Following the measurement of protein levels using a BCA kit (Beyotime), the protein specimens underwent separation through 12% sodium dodecyl sulfate-polyacrylamide gel electrophoresis (SDS-PAGE) SDS-PAGE and were subsequently moved onto polyvinylidene fluoride (PVDF) membranes (Millipore). Following a tris-buffered saline Tween-20 (TBST) wash of the membrane (20 mM Tris-HCl, 150 mM NaCl, and 0.1% Tween 20, pH 7.5), the membranes underwent an overnight incubation with primary antibodies IGF2BP3 (Abcam, ab177477, 1:1000) and GAPDH (Abcam, ab9485, 1:2500), along with the secondary antibody (Abcam, ab205718, 1:2000) for an hour at ambient temperature. Finally, the membrane was developed using ECL (Thermo Fisher) in Image Quant LAS4000 mini (GE Healthcare, UK). Protein bands were analyzed by ImageJ software.

### Xenograft Models

Animal procedures were approved by The First Affiliated Hospital of Wannan Medical College Ethics Committee (Approval No.2022-AH811; approval date: August 12, 2022). Vital River Labs (Beijing, China) provided 30 thymic BALB/c nude male mice (4-6 weeks old, 16-18 g) that were accommodated in a room adhering to Specific pathogen-free (SPF) standards, equipped with adequate water and food. MKN-45 cells transfected with sh-NC or sh-sh-has_circ_0013048 (1 × 10^7^ cells per mouse) were subcutaneously injected into mice (n = 6) to construct a subcutaneous xenograft tumor model, or injected into nude mice (n = 6) by tail vein to construct a liver metastasis model. Tumor volumes of mice were monitored weekly (volume = length × width^2^/2). Twenty-one days after injection, mice were euthanized using CO_2_, tumor tissues and liver tissues were collected, and tumors were dissected and weighed. Tumor tissues were fixed with paraformaldehyde for further study. Immunohistochemistry (IHC) analysis of IGF2BP3 and Ki67 in the tumor tissues was performed. HE staining was performed on liver tissues to observe metastatic nodules.

### Hematoxylin and Eosin Staining

Paraformaldehyde-fixed liver tissues were embedded in paraffin. The embedded liver tissue sections (4 μm) were stained with HE staining (Beyotime), followed by a 10-minute differentiation with 1% hydrochloric acid in alcohol. Following a 10-second rinse with 2% sodium bicarbonate (Beyotime), the samples underwent a 3-minute eosin staining, dehydration using a series of alcohol concentrations, permeabilization using xylene, and sealing with neutral gum. Images were observed using a microscope (Leica, Germany).

### Immunohistochemistry

Samples of tumor tissue were preserved in 4% paraformaldehyde overnight, encased in paraffin, and sectioned into 4-μm slices. The sections underwent differentiation using xylene and were sequentially rehydrated in ethanol concentrations ranging from 100% to 50%. Following the retrieval of antigens in a 10 mM citrate solution, the tissue samples underwent a 10-minute incubation in 3% H_2_O_2_ and were then sealed for an hour. Sections of tissue underwent an overnight incubation with IGF2BP3 (Invitrogen, OTI4H5) and Ki67 (Abcam, ab15580), were treated with a secondary antibody (Abcam) at 37°C for an hour, dyed with DAB substrate (Sigma-Aldrich), and visualized under a light microscope (Leica).

### Bioinformatics Analysis

For clarifying the aberrant expression patterns of circRNAs in Hepatocellular carcinoma (HCC), the GSE83521 dataset was retrieved from the circMine database (http://www.biomedical-web.com), employing differential expression analyses to examine circRNAs across the dataset comprising 6 tumors and 6 normal mucosal tissues. The starBase database (https://rnasysu.com/encori/) predicted possible targets for the RNA-binding protein of hsa_circ_0007376.

### Statistical Analysis

A total of 3 experiments were conducted independently, with over 3 samples collected each time. The results were expressed as mean ± SD. Analyses were conducted using GraphPad Prism 8.0 (GraphPad, San Diego, USA). Two- and multiple-group differences were analyzed using Student’s *t*-tests or one-way ANOVA, respectively. Based on the *χ*^2^ method, correlations between hsa_circ_0007376 and clinicopathological characteristics were determined.* P* < .05 was considered statistically significant.

## Results

### Expression and Characterization of hsa_circ_0007376 in Gastric Cancer

**Figure 1.**
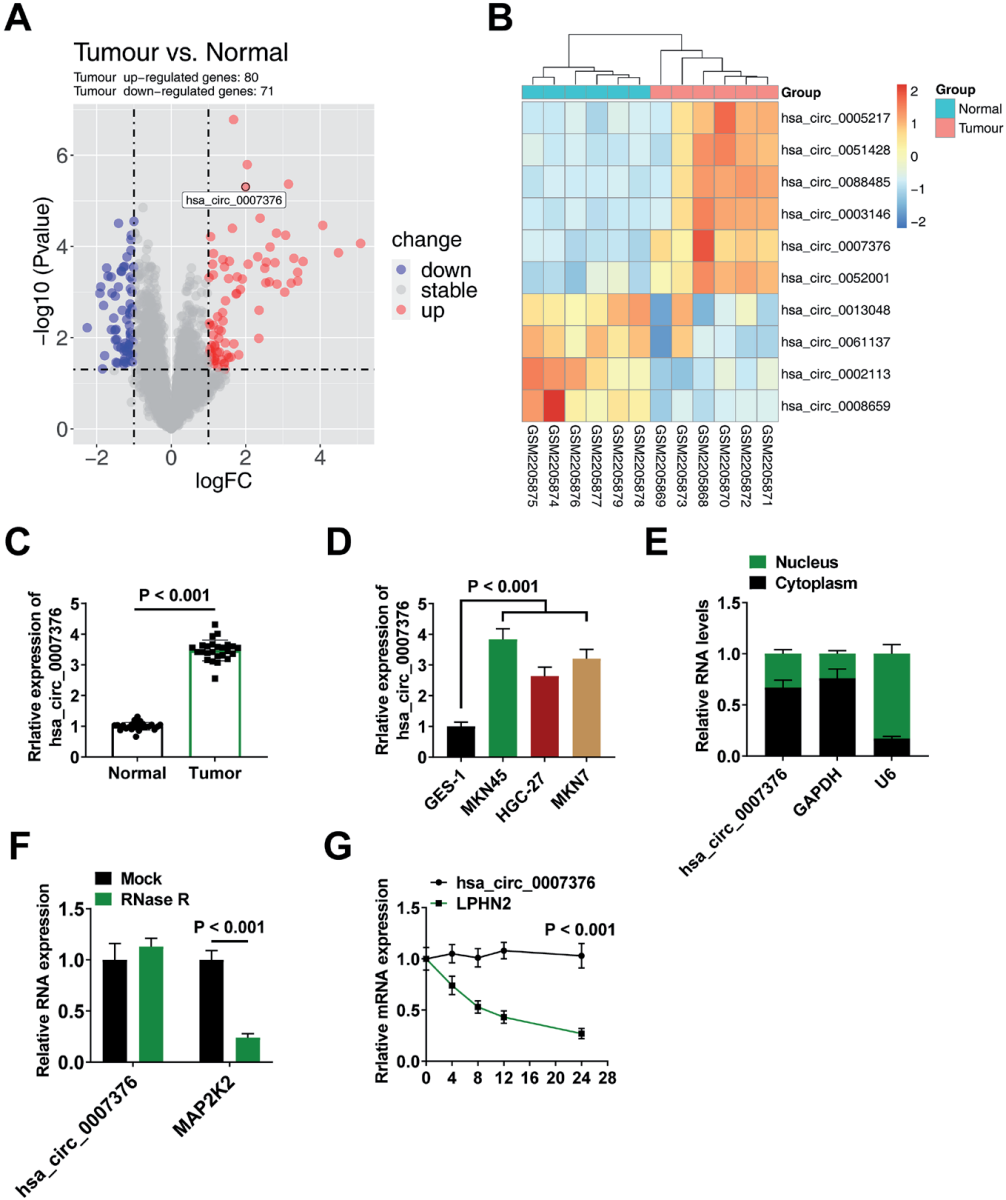
Expression and characterization of hsa_circ_0007376 in GC. A: Volcano plot of differentially expressed circRNAs (red: upregulated, blue: downregulated); B: Heatmap showing differentially expressed circRNAs; hsa_circ_0007376 was upregulated in GC tissues, row: group, column: circRNA; the color key represents the expression value of circRNA, red: highest, blue: lowest; C: Relative hsa_circ_0007376 expression levels in GC tissues and adjacent tissues determined using RT-qPCR; D: Relative hsa_circ_0007376 expression levels in GES-1 and 4 GC cell lines determined using RT-qPCR; E: Nucleoplasmic isolation assay to detect hsa_circ_0007376 expression in the nucleus and cytoplasm of cells; F: RNase R assay to verify the cyclic nature of hsa_circ_0007376; G: Actinomycin D assay to test the stability of hsa_circ_0007376. Data are shown as mean ± SD. *P < .05.

Gastric cancer-related data set GSE83521 containing 6 tumors and 6 auxiliary normal mucosal tissues was analyzed by the circMine database. A highly differentially expressed circRNA, hsa_circ_0007376, was upregulated in GC tissues compared to nearby tissues (log2|average normalized fold change| > 0.8 and *P* < .001) (Figure 1A and B). The significant upregulation of hsa_circ_0007376 in GC tissues was also confirmed in 50 matched pairs of GC and adjacent tissues (Figure 1C). hsa_circ_0007376 expression level was correlated with lymphatic metastasis, TNM stage, and tumor size; however, gender and age were not significantly correlated (Table 1). Next, RT-qPCR examined that hsa_circ_0007376 expression was increased in GC cells (MKN45, HGC-27, and MKN7) compared to GES-1 (Figure 1D). Given the high level of hsa_circ_0007376 in MKN-45 cells, subsequent studies were performed in MKN-45 cells. A nucleoplasmic separation experiment detected that hsa_circ_0007376 was mainly distributed in the MKN-45 cytoplasm (Figure 1E). MAP2K2 linear mRNA was reduced by RNase R treatment, but endogenous hsa_circ_0007376 was resistant (Figure 1F). Moreover, treatment of MKN-45 cells with the transcriptional inhibitor actinomycin D revealed that hsa_circ_0007376 showed high stability in the cells (Figure 1G).

### Gastric Cancer Cells are Repressed in Proliferation and Migration When hsa_circ_0007376 is Knocked Down

**Figure 2.**
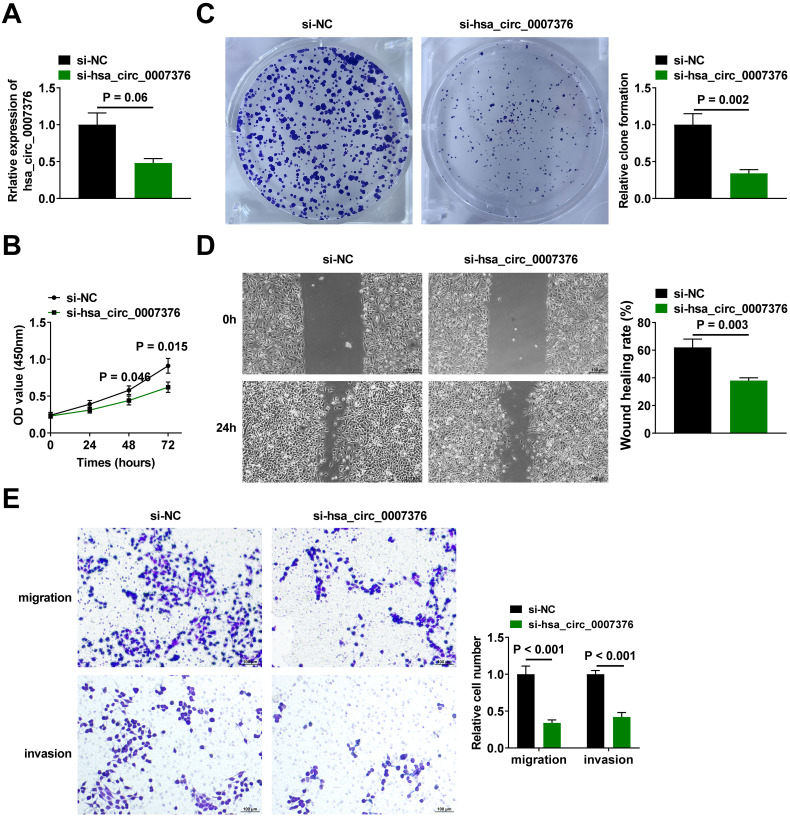
Downregulating hsa_circ_0007376 inhibits the proliferation and migration of GC cells. A: RT-qPCR to verify the knockdown effect of si-hsa_circ_0007376; B, C: CCK-8 and clone formation assay to analyze the effect of downregulating hsa_circ_0007376 on cell proliferation; D: Wound healing assay to detect the effect of downregulating hsa_circ_0007376 on MKN-45 cell migration; E: Transwell assay to analyze the effect of downregulating hsa_circ_0007376 on cell migration and invasion. Data are shown as mean ± SD. *P < .05.

For exploring the effect of hsa_circ_0007376 on the phenotype of GC cells, si-hsa_circ_0007376 was treated in MKN-45 cells to knock down hsa_circ_0007376. Quantitative reverse transcription polymerase chain reaction verified the transfection effect (Figure 2A). Subsequently, cell proliferation of MKN-45 cells with downregulating hsa_circ_0007376 was detected by CCK-8, revealing that suppressing hsa_circ_0007376 inhibited cellular proliferation (Figure 2B). Colony formation assay verified that downregulating hsa_circ_0007376 impaired cellular colony formation ability (Figure 2C). Wound healing assay and Transwell assay were conducted to investigate the effect of hsa_circ_0007376 on cell migration. The migration ability of MKN-45 cells with downregulating hsa_circ_0007376 was inhibited (Figure 2D). MKN-45 cells were inhibited from migrating and invading when hsa_circ_0007376 was downregulated in the Transwell assay (Figure 2E).

### hsa_circ_0007376 Binds to Insulin-Like Growth Factor 2 mRNA Binding Protein 3 in Gastric Cancer Cells

**Figure 3.**
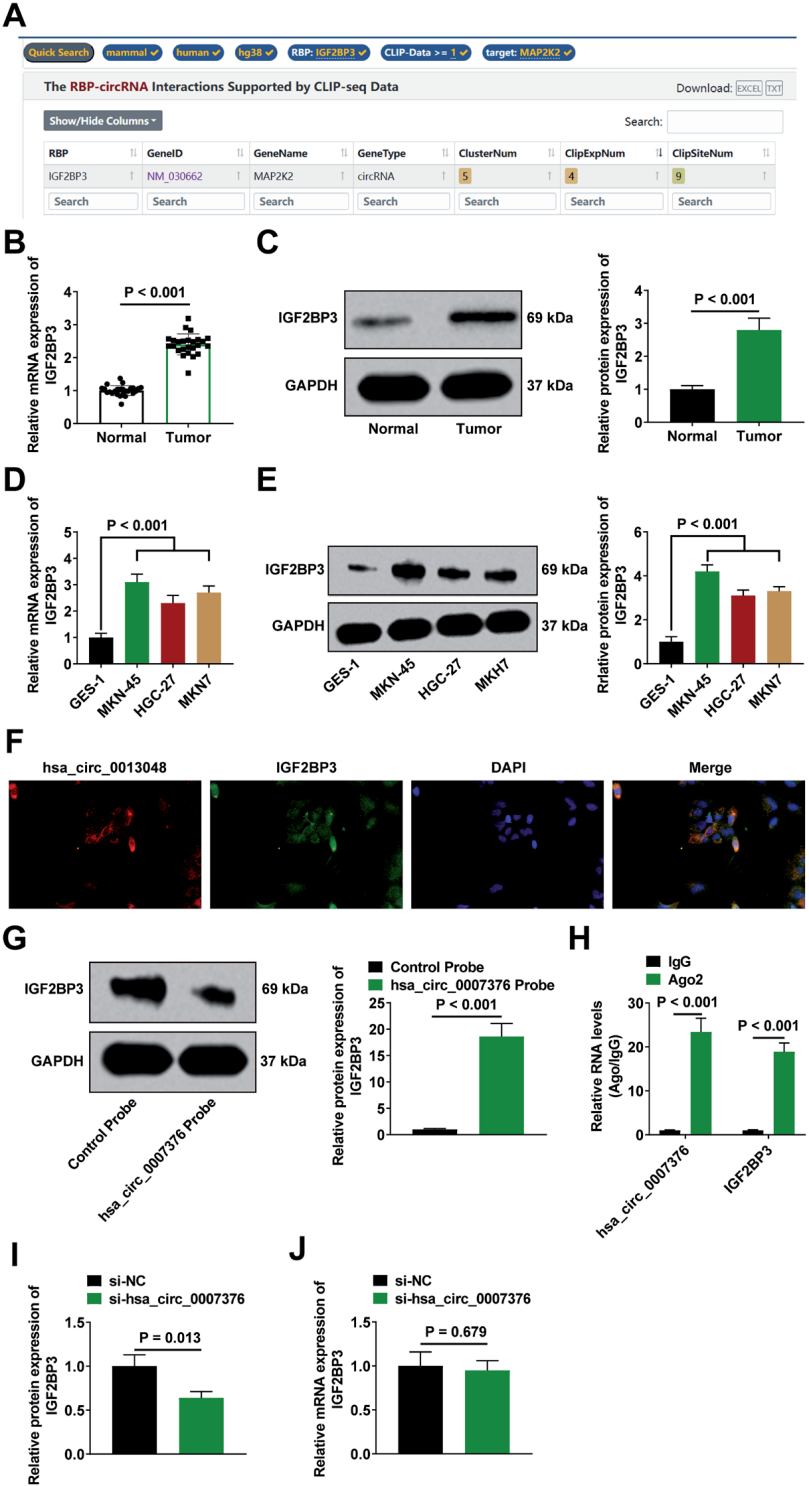
hsa_circ_0007376 binds to IGF2BP3 in GC cells. A: CircInteractome predicted the binding of IGF2BP3 to hsa_circ_0007376; B, C: RT-qPCR and Western blot to detect the expression of IGF2BP3 in GC tissues; D, E: RT-qPCR and Western blot to detect IGF2BP3 in GC cell lines (MKN45, HGC-27, and MKN7); F: Co-localization of hsa_circ_0007376 with IGF2BP3; G, H: RNA pull-down and RIP to verify the binding of hsa_circ_0007376 to IGF2BP3; I: Western blot detected the effect of knocking down hsa_circ_0007376 on IGF2BP3 protein expression; J: RT-qPCR and Western blot to detect IGF2BP3 expression in GC cell lines (MKN45, HGC-27 and MKN7). Data are shown as mean ± SD. * *P* < .05.

According to starBase (https://rnasysu.com/encori/), hsa_circ_0007376 had a binding site for IGF2BP3 (Figure 3A). Based on this information, the binding of hsa_circ_0007376 to IGF2BP3 was verified through a series of experiments. The expression of Insulin-like growth factor 2 mRNA binding protein 3 in GC tissues (Figure 3B and C) and cells (Figure 3D and E) was assessed using RT-qPCR and Western blot. Insulin-like growth factor 2 mRNA binding protein 3 was found to be highly expressed in GC tissues and GC cells. In addition, hsa_circ_0007376 co-localized with IGF2BP3 in the cytoplasm, as shown by the FISH results (Figure 3F). RNA pull-down results indicated that biotinylated hsa_circ_0007376 significantly enriched IGF2BP3 in MKN-45 cells (Figure 3G). Similarly, the RIP results confirmed the interaction between hsa_circ_0007376 and IGF2BP3 (Figure 3H). However, hsa_circ_0007376 did not regulate IGF2BP3 mRNA expression in MKN-45 cells, and downregulating hsa_circ_0007376 reduced IGF2BP3 protein expression, while having no effect on its mRNA expression (Figure 3I and J).

### Insulin-Like Growth Factor 2 mRNA Binding Protein 3 Overexpression Reverses the Inhibition of Proliferation and Metastasis of Gastric Cancer Cells by Downregulating hsa_circ_0007376

**Figure 4.**
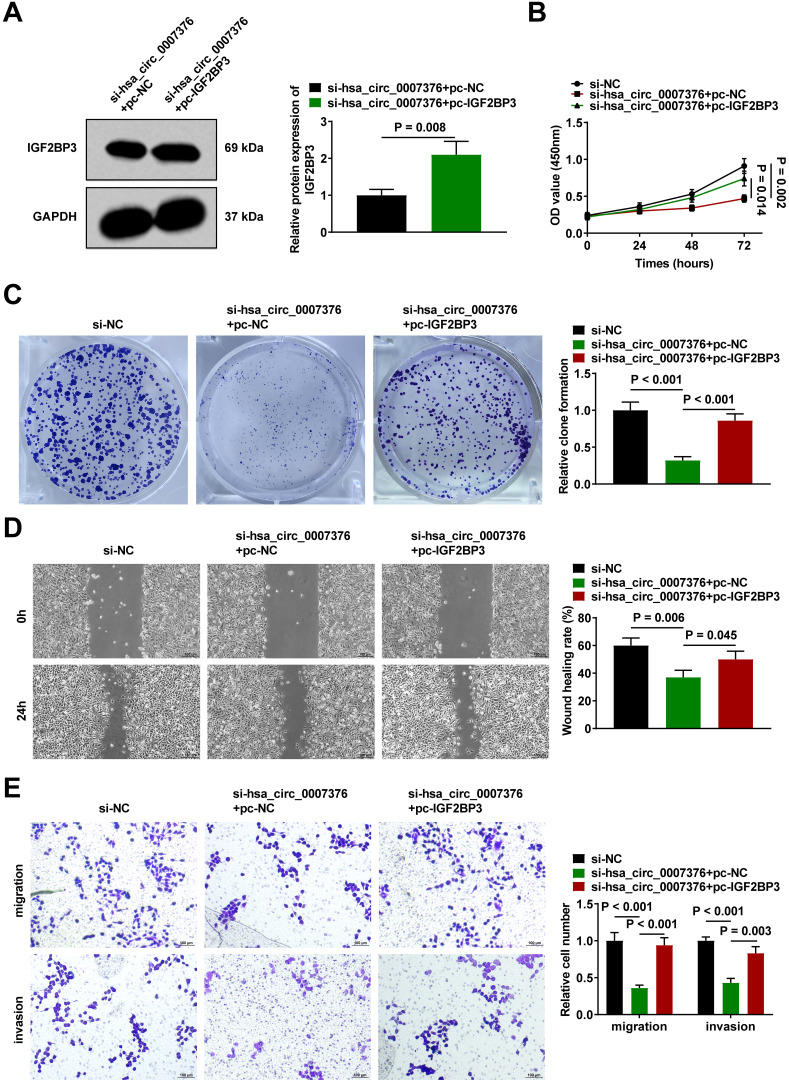
Insulin-like growth factor 2 mRNA binding protein 3 overexpression reverses the inhibition of proliferation and metastasis of GC cells by downregulating hsa_circ_0007376. A: Western blot verified the transfection efficiency of pc-IGF2BP3; B, C: CCK-8 and clone formation assay to analyze cell proliferation; D: Wound healing assay to detect MKN-45 cell migration; E: Transwell assay to analyze cell migration and invasion. Data are shown as mean ± SD. *P < .05.

hsa_circ_0007376 is capable of binding directly to IGF2BP3; however, it is not known whether this binding is responsible for regulating GC. Therefore, functional rescue experiments were conducted in MKN-45 cells by transfecting them with si-hsa_circ_0007376 alone or in combination with pc-IGF2BP3. The transfection efficiency was verified by Western blot (Figure 4A). Overexpression of IGF2BP3 reversed the action of downregulating hsa_circ_0007376 on MKN-45 cell proliferation (Figure 4B) and colony-forming ability (Figure 4C). Also, the role of downregulating hsa_circ_0007376 on MKN-45 cell migration and invasion was reversed by overexpression of IGF2BP3 (Figure 4D and E).

### hsa_circ_0007376 Promotes Gastric Cancer Metastasis In Vivo

**Figure 5.**
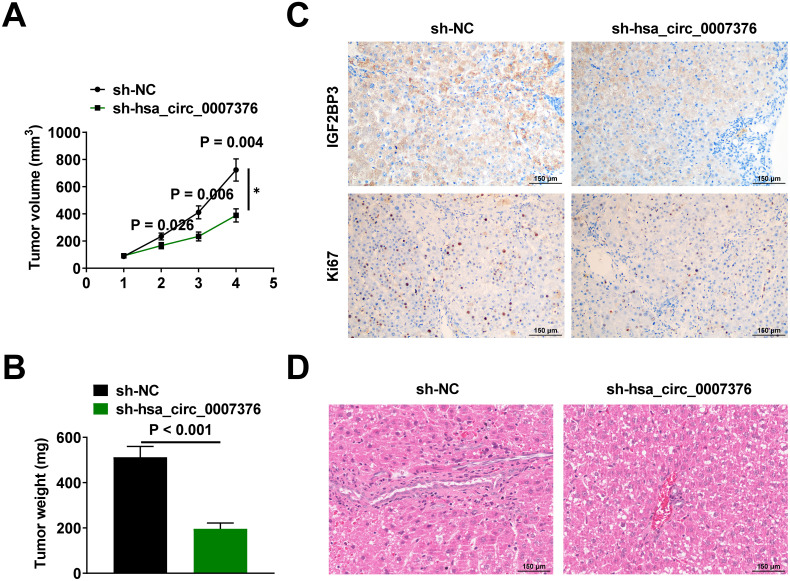
hsa_circ_0007376 promotes GC metastasis in vivo. A: Changes in the volume of mouse tumor tissues; B: Weight of mouse tumors at the modeling endpoint; C: IHC staining to detect the expression of IGF2BP3 and Ki67 in mouse tumor tissues; D: HE staining of liver tissues to observe the liver metastasis of mouse tumors. Data are shown as mean ± SD. *P < .05.

Subcutaneous injections of MKN-45 cells labeled with sh-hsa_circ_0007376 or sh-NC were made into BALB/c nude mice. Both tumor size in mice and tumor weight were reduced due to hsa_circ_0007376 downregulation (Figure 5A and B). Insulin-like growth factor 2 mRNA binding protein 3 and Ki67 levels were significantly downregulated in xenograft tumors after sh-hsa_circ_0007376 treatment (Figure 5C). To construct a mouse model of tumor metastasis, sh-NC and sh-hsa_circ_0007376 transfected MKN-45 cells were injected intravenously. HE staining was performed on the liver tissues of the mice to observe the metastatic nodules, and the degree of liver metastasis was reduced in mice after hsa_circ_0007376 downregulation (Figure 5D).

## Discussion

In GC therapy, it is imperative to examine the key targets affected by aberrant expression changes, since GC poses a greater threat to human health. It is thought that circular RNAs are involved in disease pathologies, including cancer.^6^^,^^22^ CircRNAs seem to play a key role in tumorigenesis, invasion, and metastasis, among many biological processes.^23^^-^^26^ Within GC, certain aberrantly expressed circRNAs are known to influence the biological activities of GC cells, including their proliferation, invasion, migration, cycle, and apoptosis. As an illustration, circRNA_0023685 fosters the growth and programmed cell death of GC cells in a lab setting and augments the expansion of GC tumors in a xenograft framework.^27^ circUSP1 is a novel marker linked to GC development.^28^ This study identified hsa_circ_0007376, which promotes tumor development in GC. hsa_circ_0007376 expression was upregulated in tumor tissues of GC patients and correlated with lymphatic metastasis, tumor stage, and tumor size. Consistent with previous reports, hsa_circ_0007376 localized to the cytoplasm, was more stable than and was less susceptible to RNase R degradation linear RNA. Gastric cancer cells were inhibited in proliferation, invasion, and migration by downregulating hsa_circ_0007376. The inhibition of cancer development and metastasis was also confirmed through xenograft experiments. CircRNAs may be significant actors in cancer, as evidenced by the findings supporting hsa_circ_0007376 as a potential target for GC therapy.

One way circRNAs regulate tumor biology is through interactions with proteins. circRNAs can act as RBP “super sponges” by binding to RBPs, thereby altering splicing patterns or mRNA stability.^29^ Insulin-like growth factor 2 mRNA binding protein 3 is upregulated in several cancers.^30^ Research indicates that IGF2BP3 may engage with noncoding RNAs such as miRNAs, lncRNAs, and circRNAs, impacting tumorigenesis. Specifically, increasing evidence suggests that circRNAs have the capacity to control numerous cancer-related biological activities via interactions with IGF2BP3. circNEIL3 promotes glioma progression by stabilizing IGF2BP3.^31^ circARID1A binds to IGF2BP3 in GC and promotes cancer proliferation.^21^ There are also hsa_circ_0003258-IGF2BP3^32^ and hsa_circ_0000231-IGF2BP3.^33^ It could therefore be possible to understand the oncogenic role of IGF2BP3 in GC by identifying the hsa_circ_0007376 RNA that interacts with it. In this study, the starBase database was used to predict the existence of a binding site between hsa_circ_0007376 and IGF2BP3. FISH showed that hsa_circ_0007376 was co-localized with IGF2BP3 in the cytoplasm. RIP with RNA pull-down verified the binding. In addition, downregulating hsa_circ_0007376 did not affect IGF2BP3 mRNA but was able to downregulate its protein expression. This is similar to the findings of Yang et al^16^ Functionally, the oncogenic effects of hsa_circ_0007376 involved IGF2BP3, and upregulating IGF2BP3 eliminated the impact of downregulating hsa_circ_0007376 on GC cells. However, the region of hsa_circ_0007376 that can bind to IGF2BP3 needs further study.

In conclusion, hsa_circ_0007376 is upregulated in human GC tissues. Functionally, hsa_circ_0007376 promotes tumor proliferation and metastasis. Mechanistically, hsa_circ_0007376 is able to bind to IGF2BP3 to promote GC proliferation and malignant metastasis. The efficacy of nucleic acid therapy in precisely altering both coding and noncoding target genes has garnered significant interest. Presently, there’s a growing trend in the approval or clinical testing of nucleic acid medications, encompassing siRNA drugs and antisense oligonucleotides.^34^^,^^35^ New insights into GC development are revealed by this study, as well as new targets for GC treatment. However, this study has not yet screened a suitable mRNA. In the follow-up study, the mechanism will be further improved. This study only explored the circRNA-protein interaction mechanism when probing the regulatory mechanism of hsa_circ_0007376. To discover new regulatory mechanisms, further studies on molecular sponges that act as miRNAs may be needed to identify hsa_circ_0007376’s role in the ceRNA network. Finally, this study was performed only in the MKN-45 cell line with high hsa_circ_0007376 expression in order to simplify the experimental conditions and improve efficiency; however, a single cell line may not adequately represent the complexity of the entire species or disease population due to the limitations of genetic background, phenotypic characteristics, and other factors. Therefore, the regulatory mechanism of hsa_circ_0007376 in the midst of more GC cell lines will be subsequently explored.

## Figures and Tables

**Table 1.** hsa_circ_0007376 Expression and Clinicopathological Features

Characteristics	hsa_circ_0007376 Expression		*P*
	Low (n = 25) Low Expression (<median)	High (n = 25) High Expression (≥median)	
Gender			
Male	16	12	.2545
Female	9	13	
Age (years)			
≥55	15	14	.7745
<55	10	11	
Lymph node metastasis			
Yes	9	18	.0107*
No	16	7	
Tumor size (cm)			
<5	15	8	.0470*
≥5	10	17	
Clinical staging			
I/II	19	9	.0044*
III/IV	6	16	

Indicates statistical significance.

**Table 2.** Primer Sequences

Genes	Forward Primer	Reverse Primer
hsa_circ_0007376	GCTAACTATGGTCGGACGGA	GCCCAGCTTCATCAAAACCA
MAP2K2	TGGACCTGCAGAAGAAGCTG	TCACTGTAGAAGGCCCCGTA
IGF2BP3	CCCCTCGGACCTAGAAAGT	TGATGTTCCGAATGGTGGCA
U6	CTCGCTTCGGCAGCACA	AACGCTTCACGAATTTGCGT
GAPDH	CACCCACTCCTCCACCTTTG	CCACCACCCTGTTGCTGTAG

GAPDH, Glyceraldehyde-3-phosphate dehydrogenase; IGF2BP3, Insulin-like growth factor 2 mRNA binding protein 3; MAP2K2, MAPK kinase 2.

## Data Availability

Data are available from the corresponding author on request.
